# Antifungal Activity of Difenoconazole-Loaded Microcapsules against *Curvularia lunata*

**DOI:** 10.3390/jof10080519

**Published:** 2024-07-25

**Authors:** Xiaoyu Chang, Yuyan Wang, Abbas Zain, Haibing Yu, Weidong Huang

**Affiliations:** 1 College of Resources and Environment, Anhui Science and Technology University, Chuzhou 233100, China; cy410309@163.com (X.C.); wyuyan06@163.com (Y.W.); 2 College of Agriculture, Anhui Science and Technology University, Chuzhou 233100, China; zainpbg47@gmail.com

**Keywords:** chitosan microcapsules, *Curvularia lunata*, difenoconazole, antifungal activity

## Abstract

Difenoconazole-loaded (CS-DIF) microcapsules were synthesized by encapsulating difenoconazole into biocompatible chitosan. The physical and chemical properties indicated that the encapsulation and chemical loading rates were 85.58% and 61.98%, respectively. The microcapsules exhibited prominent controlled-release and surface stability performance. The cumulative release rate was only 33.6% in 168 h, and the contact angle decreased by 11.73° at 120 s compared with difenoconazole. The antifungal activity of the CS-DIF microcapsules against *Curvularia lunata* was confirmed through observations of colony growth, in vitro and in vivo inoculation, mycelium morphology, as well as DNA and protein leakage. The antioxidant enzyme activity of superoxide dismutase, peroxidase, and catalase decreased by 65.1%, 84.9%, and 69.7%, respectively, when *Curvularia lunata* was treated with 200 μg/mL microcapsules, compared with the control in 24 h. The enzymatic activity of polyphenol oxidase decreased by 323.8%. The reactive oxygen species contents of hydrogen peroxide and superoxide anions increased by 204.6% and 164%, respectively. Additionally, the soluble sugar and soluble protein contents decreased by 65.5% and 69.6%, respectively. These findings provided a novel approach to control the growth of *C. lunata* efficiently, laying a foundation for reducing the quantity and enhancing the efficiency of chemical pesticides. The CS-DIF microcapsules exhibited a strong inhibitory effect on fungus, effectively preventing and controlling leaf spot disease and showing potential for field applications. This study might be of great significance in ensuring plant protection strategies.

## 1. Introduction

The increasing global food demand has led to a corresponding rise in the use of agrochemicals. Therefore, sustainable agricultural production systems need to be explored to strengthen the push for agriculture and food security [1,2,3]. The application of nanotechnology in agriculture has attracted considerable attention [4,5]. It effectively promotes a better transport system for agrochemicals, including fertilizers, pesticides, herbicides, fungicides, and plant growth regulators [6,7,8]. Nanoscale carriers offer several advantages, including a more powerful transport system, efficient storage, and controlled-release characteristics through packaging, wrapping, polymer, surface ions, and weak bond attachment [9,10]. Moreover, they improve stability and help prevent environmental degradation, thereby reducing chemical discharge and ecological problems [11]. At the same time, their controlled-release characteristics can limit the amount of active ingredients, thus reducing agricultural chemical waste and pollution to a minimum value [12,13,14,15]. Moreover, these nanoscale carriers can have an attachment capacity that uses molecular and conformational affinity to transfer nanoscale structures to substances in the soil, affecting plant roots or surrounding soil structures and organic matter [16,17,18].

Southern blight in corn caused by *Curvularia lunata* is a serious plant disease globally [19,20]. It occurs in Europe, North America, South America, Asia, and Africa, resulting in a sharp decline in corn yield and severe losses of up to 60% [21,22]. In the mid-1980s, it was reported in the Henan, Hebei, and Shanxi provinces in China. Leaf spot disease caused corn yield to drop by 20–50% and, sometimes, even led to crop failure [23,24]. The pathogen was mainly transmitted through the air, resulting in a sudden outbreak of the disease. The symptoms are most severe during the middle and late fertility periods. Investigations showed that the main maize varieties in the Huang-Huai-Hai region were infected by *C. lunata* to varying degrees. Hence, the spread of the disease needed to be urgently controlled to prevent further loss and damage [25].

Chitosan, a derivative of chitin, the second most abundant polysaccharide in nature, has received widespread attention globally. It is found in different types of organisms, including higher plants and vertebrates. Chitosan is used in seed handling and fungicide nanowires to protect plants against fungal infections. It can enhance plant defense mechanisms by inhibiting the growth of fungal pathogens to control or reduce disease transmission in plants [26,27,28]. It has the advantages of nontoxicity, biodegradability, biocompatibility, antibacterial activity, and antioxidant activity. On the contrary, tripolyphosphate (TPP) is a nontoxic and multivalent anion. The cross-linker is added to prepare chitosan nanoparticles (ionic gelation method) [29]. Moreover, the cationic chitosan can use electronic forces to interact with the anionic TPP. Otherwise, TPP can control the nanoparticle size and nanoparticle-loading efficiency [30,31].

Furthermore, several previous studies reported on the development of fungicide transport systems using polymers, β-cyclodextrins, silica, and chitosan as carrier systems. Chitosan nanoparticles can serve as efficient nanocarriers [32]. Difenoconazole is a broad-spectrum triazole fungicide and the active ingredient in many commercial fungicides. It belongs to the class of 14 α-demethylation inhibitors [33]. In addition, it is well absorbed by plant roots and transported to other parts of the plant via xylem tissues. Diphenyl ether conazole has better disease prevention effects and hence is used more in agriculture compared with other fungicides [34,35]. Usually, a spray or package [36] can also be directly applied to the root.

In addition, dual encapsulation has been proven to increase drug release and bioavailability, but the formulation might be challenging because of drug incompatibility, which could give rise to complications [37]. Morell et al. [38] synthesized chitosan–polyvinylalcohol (PVA) particles to study the compound release mechanism of the active ingredient under various conditions. Sinha and Mitra et al. [39,40] studied the correlation between matrix erosion and release kinetics of indomethacin-loaded chitosan microspheres. Release kinetics were correlated with the concentration of chitosan in the microsphere and the pH of the release medium. Interestingly, several works have described the mechanism of nanoparticle formation through ionic gelation well [41,42]. It has been suggested that all ionic groups of TPP participated in interactions with chitosan amine groups. Based on existing research, this experiment utilized chitosan-loaded difenoconazole to prepare nanomicrocapsules with the aim of disease control and environmental pollution reduction. This approach effectively reduces the use of pesticides while increasing efficiency.

In this study, chitosan difenoconazole (CS-DIF)-loaded microcapsules were prepared using a chemical approach. Several analytical techniques, such as transmission electron microscopy (TEM), scanning electron microscopy (SEM), Fourier-transform infrared spectroscopy (FTIR), and zeta potential, were applied to characterize microcapsules. The encapsulation rate, chemical loading rate, slow-release, and contact angle were used to determine the physical and chemical properties of microcapsules. The synergistic antifungal activity of fungicide-loaded microcapsules against *C. lunata* was estimated using in vitro and in vivo approaches. The physiological and biochemical influences of microcapsules on *C. lunata*, including antioxidant enzymes, phenolic metabolism enzymes, reactive oxygen, soluble sugar, and soluble protein, were also determined. The use of fungicide-loaded microcapsules might serve as an efficient strategy to control plant pathogens, thus helping reduce the use of chemical pesticides.

## 2. Materials and Methods

### 2.1. Reagents and Fungal Isolate

Chitosan (90% deacetylation, CAS: 9012-76-4, MACKLIN, Shsnghai, China). Sodium tripolyphosphate (TPP), Tween-20, and acetic acid were purchased from Sigma-Aldrich (Saint Louis, MO, USA). Difenoconazole was purchased from Syngenta Nantong Crop Co., Ltd. (Nantong, China) *C. lunata* was preserved at the Plant Protection Laboratory of Anhui Science and Technology University.

### 2.2. Preparation of CS-DIF Microcapsules

The CS-DIF microcapsules were prepared as previously described with some modifications [43]. 0.5 g chitosan was dissolved in 100 mL of 1.0% (*v*/*v*) acetic acid, and 1 g difenoconazole was added with continuous stirring to obtain a homogenous solution. Then, 2% (*v*/*v*) Tween-20 was added to avoid particle aggregation. Finally, TPP (20 mg/mL) prepared with deionized water was added into the homogenous solution dropwise at a volume ratio of 1:2. The suspension was centrifuged at 11,000 rpm for 15 min, and the precipitate was washed with deionized water under the same centrifugation conditions. The conjugates thus obtained were freeze-dried and stored at 4 °C for further analysis.

### 2.3. Characterization of CS-DIF Microcapsules

TEM (JEM-2100F, Jeol, Hitachi, Japan) was used to analyze the morphology and diameter of particles. An SEM (S4800, Hitachi, Japan) was used to determine particle disparity. The species and elemental content of particles were verified using energy-dispersive X-ray spectroscopy (EDX). X-ray diffraction (XRD) was applied to determine the crystalline structure of synthesized particles at the 2*θ* range of 20–80°. Fourier transform infrared spectrometer (FTIR) was used to determine the functional groups on the surface of synthesized particles in the range of 4000–500 cm^−1^. The surface charge of particles was detected using a zeta potentiometer.

### 2.4. Physical and Chemical Properties of CS-DIF Microcapsules

#### 2.4.1. Encapsulation Efficiency and Fungicide Loading Capacity

Different concentrations (10, 20, 25, 50, 100, and 200 μg/mL) of difenoconazole were prepared using ethanol. The UV-Visible spectrometer of each concentration were measured at 230 nm. The standard curve of difenoconazole was drawn according to the concentration and absorption. Then, 10 mL of the CS-DIF microcapsules were centrifuged at 11,000 rpm for 15 min, and 2 mL of the supernatant was diluted five times with ethanol to determine the encapsulation efficiency. The OD_230_ of the diluted supernatant was analyzed, and the difenoconazole content in the supernatant was calculated according to the standard curve. The difenoconazole content encapsulated in microcapsules (A, mg), encapsulation efficiency (EE, %), and loading capacity (LC, %) were calculated using the following equations:

(1) A=(0.0124×L+0.4527)×5

(2) $$EE(\%)=\frac{A}{m_{1}}\times 100$$

(3) $$LD(\%)=\frac{A}{A+25+m_{2}}\times 100$$

where *L* is the amount of difenoconazole in the supernatant, mg; *m*_1_ is the content of difenoconazole, mg; and *m*_2_ is the total amount of chitosan, mg.

#### 2.4.2. Controlled Release Performance

A dialysis tubing (molecular weight: 8000–14,000 Da) was used to determine the release performance of difenoconazole from CS-DIF microcapsules at 25 °C during 0.5–168 h. 0.2 g microcapsules, supplemented with 10 mL of 70% ethanol, were placed in a dialysis membrane with closed clips at both ends. Then, the solution was mixed with 100 mL of 70% ethanol at 150 rpm with continuous stirring. The OD_230_ of the mixture was measured at each time interval, and the difenoconazole content released from the dialysis membrane was determined according to the standard curve. The acumulative release percentage (ARP, %) was calculated using the following equation:

(4) $$ARP\left(\%\right)=\frac{C\times \frac{V}{10}\times 15}{m_{3}}\times 100$$

where C is the concentration of difenoconazole solution at different time points, mg/L; V is the volume of the solution, L; and *m*_3_ is the difenoconazole content in the drug carrier system, mg.

#### 2.4.3. Wettability on Maize Leaves

The maize leaves were washed thoroughly and left to dry. The chitosan, difenoconazole, and CS-DIF microcapsule (200 μg/mL) suspension were prepared using water as the control. One drop of each solution and water were dropped onto the surface of maize leaves using a micro syringe. The change in droplet contact angle in 120 s was determined using a contact angle measuring instrument (SL200KB, Kino Industries, New York, NY, USA).

### 2.5. Antifungal Effect of CS-DIF Microcapsules on Biological Characteristics

#### 2.5.1. Colony Growth Inhibition

*C. lunata* cultured on potato dextrose agar medium for 7 days was selected. Several fungal disks (*φ* = 8 mm) were drilled around the Petri dish. The CS-DIF microcapsules were added to potato dextrose medium to achieve the final concentrations of 10, 20, 50, 100, and 200 μg/mL. Simultaneously, the medium containing 200 μg/mL chitosan and 200 μg/mL difenoconazole was also prepared. The medium with sterile water was set as the control. Finally, one fungal disk was inoculated in the center of each Petri dish and incubated at 28 °C for 5–7 days. The inhibition rate of colony growth was calculated using the following equation:

(5) Inhibition rate(%)=[(φcontrol−φtreatment)/(φcontrol−φdisc)]×100%

#### 2.5.2. Effect of Mycelium Morphology

*C. lunata* cultured for 7 days was diluted with sterile water and put into a sterile tube to detect the effect of mycelial morphology. Chitosan, difenoconazole, and CS-DIF microcapsules were added to the tube separately to achieve a concentration of 200 μg/mL. The suspension with an equal volume of sterile water was set as control. Then, the tubes were incubated at 25 °C for 72 h, and the mycelial morphology was observed under a microscope.

#### 2.5.3. Influence on DNA and Protein Leakage

The antifungal effect against *C. lunata* was also measured in terms of DNA and protein leakage. The conidia suspension of *C. lunata* was diluted with sterile water. Chitosan (200 μg/mL), difenoconazole (200 μg/mL), and CS-DIF microcapsules (10, 20, 50, 100, and 200 μg/mL) were prepared in different sterile flasks. The DNA and protein leakage contents from *C. lunata* were determined by measuring the absorbance at 260 nm (*A*_260_) and 280 nm (*A*_280_) with a UV-vis spectrometer after incubation at 28 °C for 48 h.

#### 2.5.4. Influence on In Vitro Inoculation

The maize leaves grown for about 30 days were cut, rinsed with running water, and then soaked in 5% NaClO solution and 70% alcohol for 1 and 2 min. Finally, they were rinsed with sterile water two to three times to remove the residual chemicals on the leaf surface and dried on a clean bench. The air-dried leaves were placed in Petri dishes, and several drops of the mixture containing conidia suspension (10^6^/mL) were placed on the leaves. Then, 200 μg/mL chitosan, difenoconazole, and CS-DIF microcapsules were dripped on each leaf separately. The treated and control (conidia suspension only) leaves were incubated at 25 °C for 48–72 h, and the morphological and microscopic changes were detected.

### 2.6. Physiological and Chemical Activities

#### 2.6.1. Influence on Antioxidant Enzymes

*C. lunata* cultured in potato agar liquid medium for 5 days was collected, and 1 g mycelium was treated with 200 μg/mL chitosan, difenoconazole, and CS-DIF microcapsules at 28 °C for 0, 3, 6, 12, and 24 h. The activities of antioxidant enzymes, including superoxide dismutase (SOD), peroxidase (POD), and catalase (CAT) were measured using the corresponding kits from Nanjing Jisi Huiyuan Biological Co., Ltd. (Nanjing, China).

#### 2.6.2. Influence on Phenol Metabolism Enzymes

The activities of phenol metabolism enzymes of phenylalnine ammonialyase (PAL) and polyphenol oxidase (PPO) were measured using the corresponding kits from Nanjing Jisi Huiyuan Biological Co., Ltd.

#### 2.6.3. Influence on Active Oxygen Components

The reactive oxygen components of hydrogen peroxide (H_2_O_2_) and superoxide anion (O_2_^−^) were measured using the corresponding kits from Nanjing Jisi Huiyuan Biological Co., Ltd.

#### 2.6.4. Influence on Soluble Sugar and Protein Contents

The soluble sugar and protein contents were measured using the corresponding kits from Nanjing Jisi Huiyuan Biological Co., Ltd.

### 2.7. Statistical Analysis

IBM SPSS software 24.0 was employed to perform statistical analysis. The mean standard error from three biological replicates is presented in the dataset. Duncan’s multiple-range tests were carried out using a one-way analysis of variance (ANOVA) to identify significant differences among the different experimental groups. Distinct letters are used to denote statistically significant differences, indicating a significance level of *p* < 0.05.

## 3. Results and Discussion

### 3.1. Characterization of CS-DIF Microcapsules

**Figure 1:**
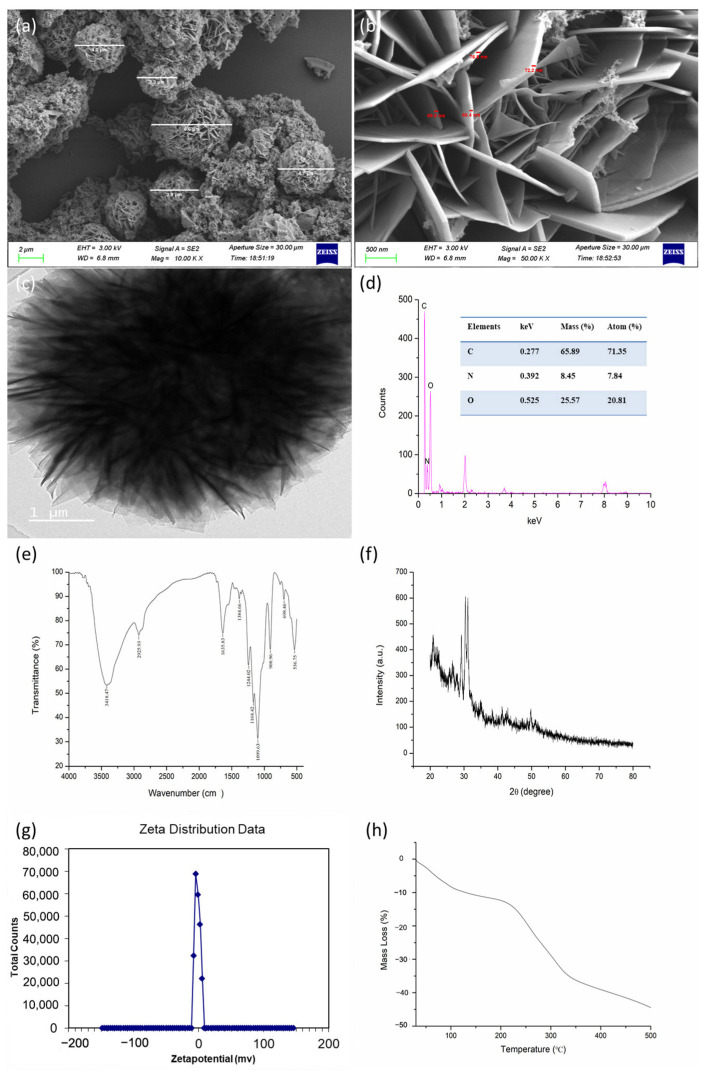
Characterization of CS-DIF microcapsules. (**a**,**b**) SEM images with different magnification; (**c**–**h**) TEM, EDX, FTIR, XRD, zeta potential, and TG.

The SEM and TEM images revealed that the synthesized CS-DIF microcapsules were shaped similarly to flower petals. The diameter was in the range of 3.3–6.6 μm, and the flake thickness of the particles was between 60.4 and 76.9 nm (Figure 1a–c). The morphology and particle size were influenced by factors such as synthesis mode, synthetic materials, and synthesis conditions [44,45]. As mentioned earlier, Tween-20 was added as a stabilizer [46]. Tween-20 could reduce the surface tension, stabilize the droplet phase, and prevent aggregation during the production of nanoparticles [47,48,49]. The EDX spectrum showed that the prepared microcapsules had three obvious absorption peaks at 0.277, 0.392, and 0.525 keV, corresponding to the C, N, and O elements, respectively. The mass ratios of the C, N, and O elements were 65.89%, 8.45%, and 25.57%, respectively. The atomic ratios of the C, N, and O elements were 71.35%, 7.84%, and 20.81%, respectively (Figure 1d). The FTIR spectrum of the synthesized CS-DIF microcapsules in the range of 4000–500 cm^−1^ is shown in Figure 1e. The bands at 3418.47 and 1635.83 cm^−1^ were attributable to the enhanced hydrogen bonding and electrostatic interactions between the chitosan amino and TPP phosphate groups in the chitosan carrier. The band at 3418.47 cm^−1^ corresponded to the OH group of difenoconazole, the band at 2925.93 cm^−1^ corresponded to the alkanyl group (C–H), and the band at 1635.83 cm^−1^ represented the CO–NH_2_ group. Further, the band at 1383.42 cm^−1^ was attributable to the stretching and bending vibrations of C–H and the bands at 1244.02 and 1168.42 cm^−1^ were attributable to the ether and amino groups, respectively. The observed vibrations at 1099.63, 908.96, 698.46, and 536.75 cm^−1^ confirmed the presence of native phosphate. The additional band of difenoconazole was attributable to the C–N stretch, C=C bonding, and C–Cl stretch. These findings demonstrated that difenoconazole was wrapped in the chitosan matrix. The XRD pattern is considered the fingerprint of periodic atomic arrangements in a given material [50]. As shown in Figure 1f, the maximum diffraction peaks appeared at 29.26°, 30.41°, and 31.15°, corresponding to its crystal structure. As shown in Figure 1g, the zeta potential analysis indicated that the synthesized microcapsules were positively charged, with a value of 5.14 mV. The positive charge could contribute to their dispersion, stability, and good colloidal properties [51,52]. The thermal properties of the synthesized CS-DIF microcapsules were determined by thermogravimetric (TG) analysis. The microcapsules displayed thermally stable performance in the air. The mass loss was in the range of 0.13–44.44% when the temperature was increased from 30 °C to 500 °C.

### 3.2. Physical and Chemical Properties of CS-DIF Microcapsules

#### 3.2.1. Encapsulation Efficiency and Drug Loading Capacity

**Figure 2:**
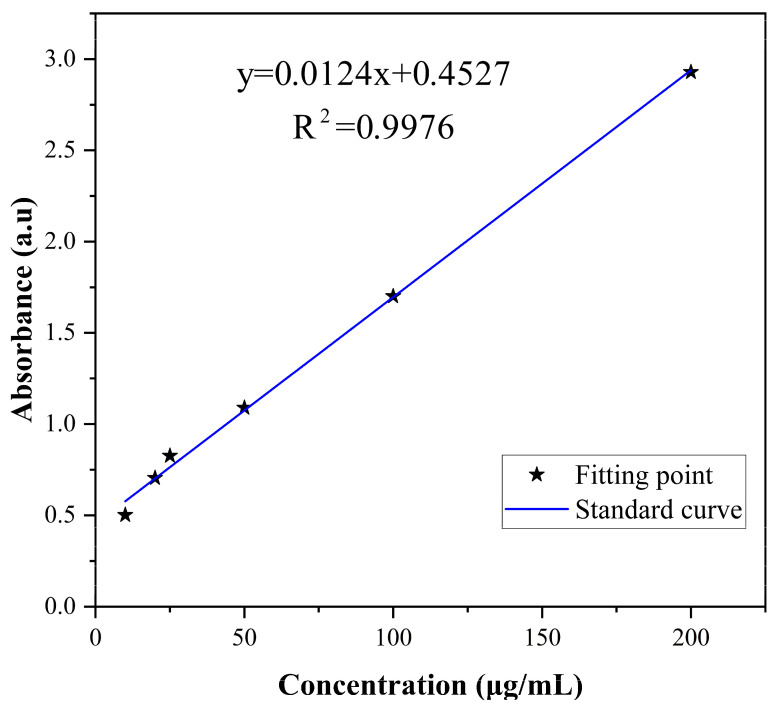
Standard curve of difenoconazole.

The corresponding relation between the concentration and absorbance of difenoconazole is shown in Figure 2. The standard curve was *y* = 0.0124*x* + 0.4527, and the correlation coefficient *R*^2^ = 0.9976, indicating that difenoconazole had a good linear correlation with absorbance in the range of 5–200 μg/mL. EE and LD were calculated as 85.58% and 61.98%, respectively, using the standard curve and the equations of EE and LD. As shown in the literature, larger particles provide higher drug encapsulation efficiency [53].

#### 3.2.2. Controlled-Release Performance

**Figure 3:**
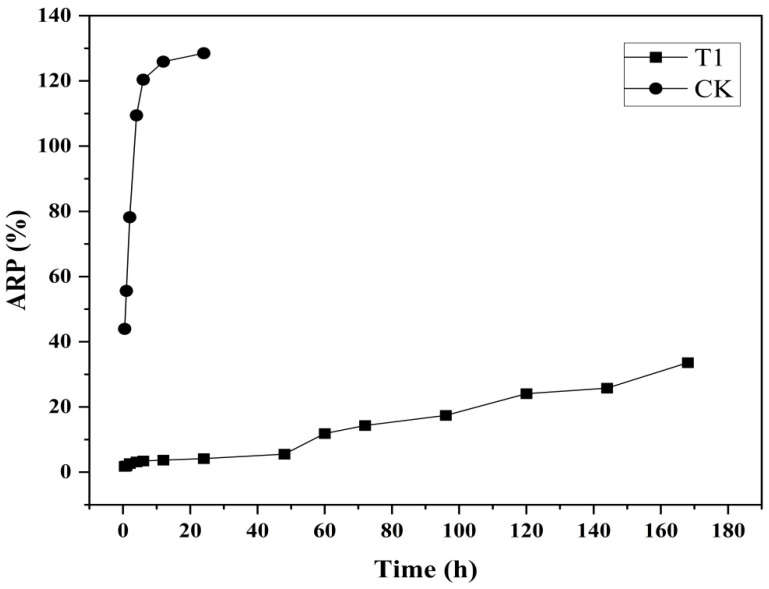
Controlled release performance of difenoconazole (CK) and CS-DIF microcapsules (T1).

As shown in Figure 3, the synthesized CS-DIF microcapsules exhibited prominent controlled-release performance compared with the control. Difenoconazole (CK) alone released 43.93% in 0.5 h, and the ARP reached 109.46% after incubating for 4 h. However, the CS-DIF microcapsules (T1) exhibited obvious controlled-release characteristics during the slow-release stage from 0.5 to 48 h. The release rate was in the range of 1.8–5.53% and increased from 11.87% to 33.6% in the rapid-release stage from 60 to 168 h. This was similar to previous findings, indicating that the release time of fungicides was much longer compared with that of hexaconazole (130 h) and dazomet (50 h). On encapsulation into chitosan nanoparticles, the cumulative release rates were 93.6% and 76.4% [54].

#### 3.2.3. Wettability Performance on Maize Leaves

**Figure 4:**
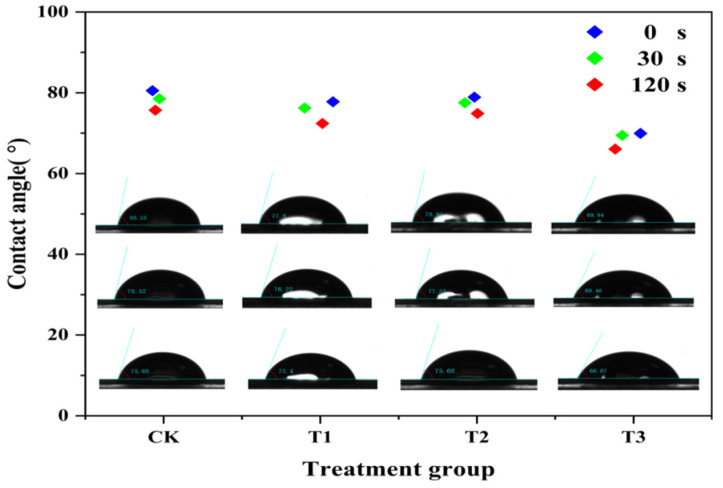
Leaf surface contact angle of the water (CK), chitosan (T1), difenoconazole (T2), and CS-DIF microcapsules (T3) on the controlled-release performance of CS-DIF microcapsules.

The wettability performance of 200 μg/mL chitosan (T1), difenoconazole (T2), and CS-DIF microcapsules (T3) on maize leaves were compared in terms of contact angle. As shown in Figure 4, the contact angle of all the treatments and water (CK) decreased gradually with time. In the range of 0–120 s, the contact angle of CK decreased from 80.53° to 75.68°. The T1 decreased from 77.8° to 72.4°, T2 decreased from 78.91° to 75.68°, and T3 decreased from 69.94° to 66.07°. The contact angle of T2 was similar to that of CK (75.68°), whereas the contact angle of T1 and T3 decreased by 3.28° and 9.61° in 120 s, respectively. The result demonstrated that CS-DIF microcapsules could effectively reduce the contact angle of drops on maize leaves and enhance their support of the leaf surface. Rotenone-loaded mesoporous silica nanoparticles (RotMSNS) could significantly decrease the contact angle from 83° to 64°, improving the support of the leaf surface compared with treatments with rot suspension or water [55].

### 3.3. In Vitro Antifungal Activity against C. lunata

#### 3.3.1. Influence on Inhibition Rate

**Figure 5:**
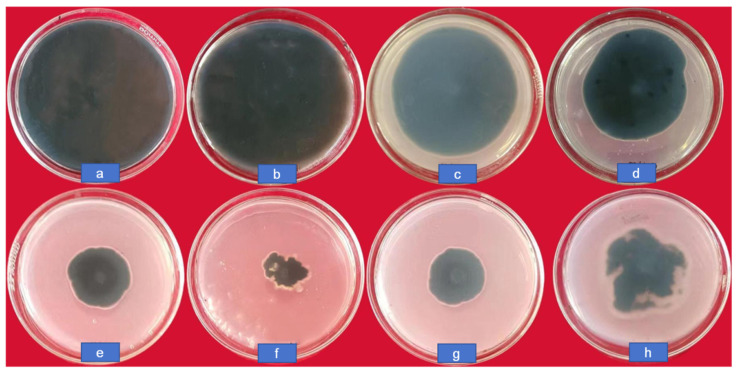
Colony growth of *C. lunata* treated with CS-DIF microcapsules, difenoconazole, and chitosan. (**a**) control, (**b**–**f**) represent the concentration of CS-DIF microcapsules was at 10, 20, 50, 100, and 200 μg/mL, respectively, (**g**) 200 μg/mL difenoconazole, (**h**) 200 μg/mL chitosan.

The colony growth of *C. lunata* at different concentrations (0, 10, 20, 50, 100, and 200 μg/mL) of CS-DIF microcapsules, difenoconazole (200 μg/mL), and chitosan (200 μg/mL) is shown in Figure 5. The diameter in the control was 8.65 cm; it decreased gradually along with an increasing concentration of CS-DIF microcapsules. It was only 2.2 cm when the concentration was 200 μg/mL, and the inhibition rate reached 82.16%. At the same concentration, the colony diameters with difenoconazole and chitosan were 3.35 and 4.1 cm, respectively. The inhibition rates were 67.51% and 57.96%, respectively. This demonstrated that the CS-DIF microcapsules could enhance the antifungal effect of difenoconazole and chitosan.

#### 3.3.2. Influence on Mycelium Morphology

**Figure 6:**
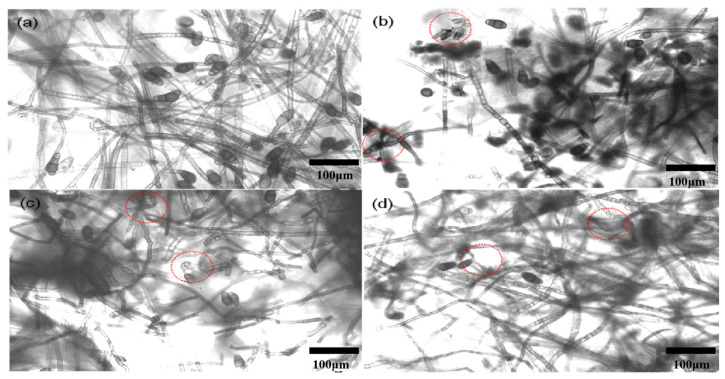
Mycelium morphology of *C. lunata* treated with sterile water (**a**), 200 μg/mL CS-DIF microcapsules (**b**), 200 μg/mL difenoconazole (**c**), and 200 μg/mL chitosan (**d**) influence of mycelium morphology.

The mycelium morphology of *C. lunata* treated with 200 μg/mL CS-DIF microcapsules, difenoconazole, and chitosan is shown in Figure 6. For the control, the mycelium was intact and had a smooth appearance (Figure 6a). However, it exhibited different degrees of damage, such as distortion and deformation, when treated with 200 μg/mL difenoconazole (Figure 6c) and chitosan (Figure 6d). In particular, the mycelium treated with 200 μg/mL CS-DIF microcapsules was obviously broken and disintegrated (Figure 6b). The abnormal change in mycelium was labeled with dotted lines and circles.

#### 3.3.3. Effect on DNA and Protein Leakage

**Figure 7:**
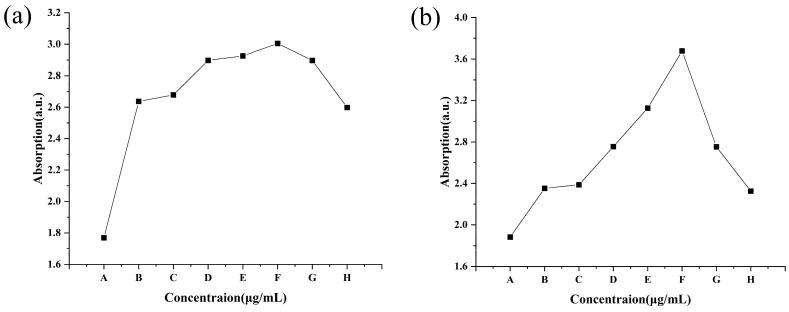
Leakage of nucleic acids (**a**) and protein (**b**) from *C. lunata*. (A) Control; (B–F) 10, 20, 50, 100, and 200 μg/mL of CS-DIF microcapsules; (G) 200 μg/mL difenoconazole; and (H) 200 μg/mL chitosan.

The DNA and protein of *C. lunata* obviously leaked after treatment with different concentrations of CS-DIF microcapsules, difenoconazole, and chitosan for 24 h. The *A*_260_ for the control was 1.77, while it increased from 2.64 to 3.01 with an increase in the concentration of CS-DIF microcapsules from 10 to 200 μg/mL. The increase was between 49.15% and 70.06% compared with the control. The *A*_260_ values of 200 μg/mL difenoconazole and chitosan were 2.90 and 2.60, and the increases were 63.84% and 46.89%, respectively (Figure 7a). At the same concentration, the CS-DIF microcapsules induced the leakage of more DNA from the mycelium compared with difenoconazole (6.22%) and chitosan (23.17%). The *A*_280_ for the control was 1.88, whereas it increased from 2.35 to 3.68 with an increase in the concentration of CS-DIF microcapsules from 10 to 200 μg/mL. The increase was between 25.00% and 95.74% compared with the control. The *A*_280_ values of 200 μg/mL difenoconazole and chitosan were 2.75 and 2.33, and the increases were 46.28% and 23.94%, respectively (Figure 7b). The CS-DIF microcapsules also induced the leakage of more protein from the mycelium compared with difenoconazole (49.46%) and chitosan (71.80%) at the same concentration.

#### 3.3.4. Effect on In Vitro Inoculation of Maize Leaves

**Figure 8:**
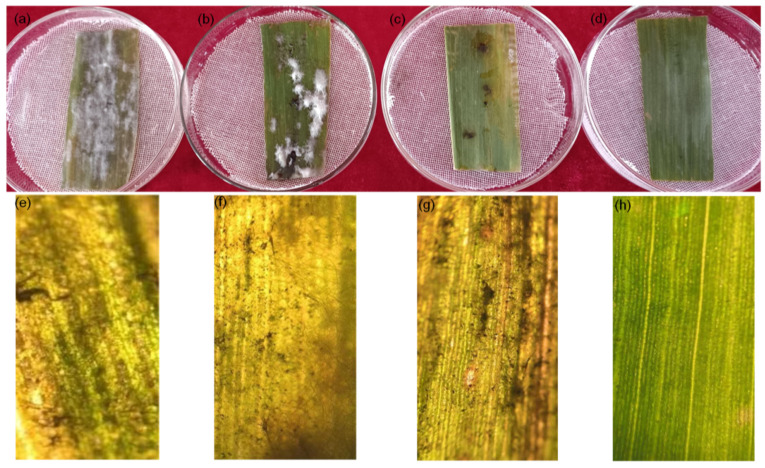
Influence of in vitro inoculation on detached maize leaves. (**a**) Control strain; (**b**–**d**), 200 μg/mL chitosan, difenoconazole, and CS-DIF microcapsules; (**e**–**h**), local microscopic images of (**a**–**d**) with a microscopic magnification of 100×.

Figure 8 shows the effects of chitosan (Figure 8b), difenoconazole (Figure 8c), and CS-DIF microcapsules (Figure 8d). The detached maize leaves inoculated with *C. lunata* were treated with the same concentration (200 μg/mL) compared with the control (Figure 8a). Other treatments reduced the degree of infection of pathogens in detached leaves to varying degrees, among which the effect of the CS-DIF microcapsules was the most obvious. The mycelium was not evident on the surface of the leaves. The color was similar to that of leaves without inoculation. The microscopic imaging results further confirmed that chitosan (Figure 8f) and difenoconazole (Figure 8g) could restrict the expansion of pathogens in detached leaves to a certain extent, but the effect was not obvious. The CS-DIF microcapsules significantly inhibited the infection and expansion of pathogens in the detached leaves and exerted a strong synergistic antifungal effect in vitro (Figure 8h).

### 3.4. Biochemical Influence against C. lunata

#### 3.4.1. Antioxidant Enzyme Activity

**Figure 9:**
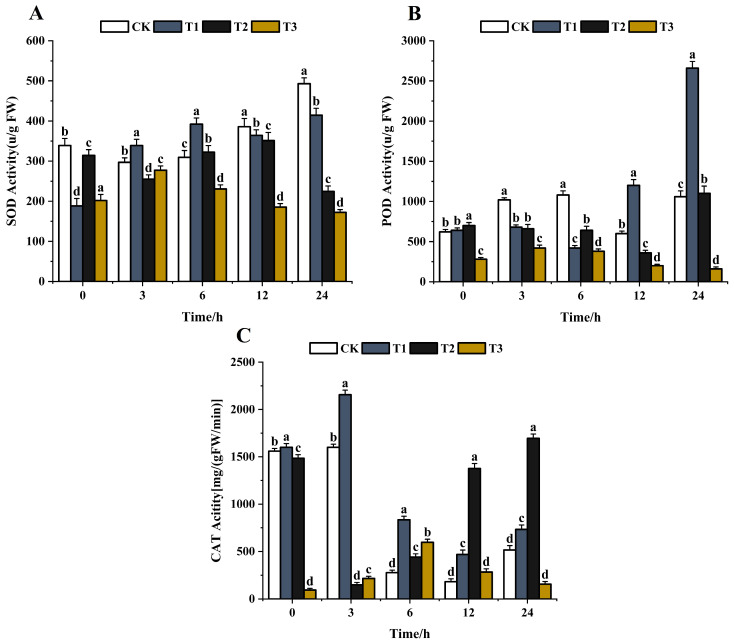
Influence on antioxidant enzyme activity. (**A**) SOD; (**B**) POD; and (**C**) CAT. T1–T3 represent 200 μg/mL chitosan, difenoconazole, and CS-DIF microcapsules, respectively. Significant differences were determined using one-way ANOVA and Duncan’s multiple range test, as indicated with different letters in the same column of different groups at the *p* < 0.05 significance level.

After stimulation with foreign substances, pathogens can produce SOD, POD, CAT, and other antioxidant enzymes to reduce the damage [56]. The lower the activity of antioxidant enzymes, the more serious the harm. SOD is the first detoxification enzyme and the most powerful antioxidant in the cell [57,58]. The effects of the same concentration (200 μg/mL) of chitosan, difenoconazole, and CS-DIF microcapsules on the antioxidant enzyme activities of *C. lunata* (0–24 h) are shown in Figure 9. With the extension of treatment time, the SOD activity of the control strain gradually increased and peaked at 24 h. The SOD activity increased first and then decreased after chitosan treatment and decreased by 15.9% compared with the control in 24 h. The SOD activity after difenoconazole treatment showed a decreasing trend, and the activity decreased by 54.5% compared with the control in 24 h. The SOD activity increased first and then decreased after treatment with CS-DIF microcapsules and decreased by 65.1% compared with the control in 24 h (Figure 9A). The results showed that chitosan, difenoconazole, and CS-DIF microcapsules enhanced the antibacterial activity by reducing the SOD activity. The CS-DIF microcapsules had the greatest effect. The POD activity of the control strain increased with the extension of treatment time. A similar phenomenon occurred with catalysis of the oxidation of phenols and amines by hydrogen peroxide (POD), which had the dual effects of eliminating the toxicity of hydrogen peroxide and phenols and amines [59,60]. First, the POD activity of both chitosan- and difenoconazole-treated strains decreased first and then increased. Second, the POD activity increased by 150.9% and 3.8% in 24 h compared with the control strain. After treatment with CS-DIF microcapsules, the POD activity decreased by 84.9% in 24 h compared with the control. The CS-DIF microcapsules could significantly reduce the POD activity, whereas chitosan and difenoconazole had no significant effect on the activity (Figure 9B). With the extension of treatment time, the CAT activity of the control strain and the chitosan-treated strain exhibited a decreasing trend. The enzyme activity of the control strain increased by 42% in 24 h after chitosan treatment. After difenoconazole treatment, the CAT activity decreased first and then increased. The enzyme activity increased by 228.9% compared with the control in 24 h. After treatment with CS-DIF microcapsules, the CAT activity increased first and then decreased; it decreased by 69.7% compared with the control in 24 h (Figure 9C). CAT is a ROS-scavenging enzyme present in all plants, where it functions to catalyze the hydrogen peroxide into H_2_O and O_2_ in an energy-efficient manner [61] by preventing excessive H_2_O_2_ build-up and allowing important cellular processes to occur. The results showed that the CS-DIF microcapsules could enhance the antifungal activity by reducing the CAT activity. Neither chitosan nor difenoconazole decreased the CAT activity at the same time. Therefore, the inhibitory effects of 200 μg/mL chitosan and difenoconazole on pathogenic bacteria were mainly reproduced in influencing the SOD activity of the pathogen. However, the CS-DIF microcapsules displayed significant inhibitory effects on various antioxidant enzymes such as SOD, POD, CAT, and so forth. The activity results of antioxidant enzymes were consistent with their strong inhibitory activities.

#### 3.4.2. Phenolic Metabolism Enzyme Activity

**Figure 10:**
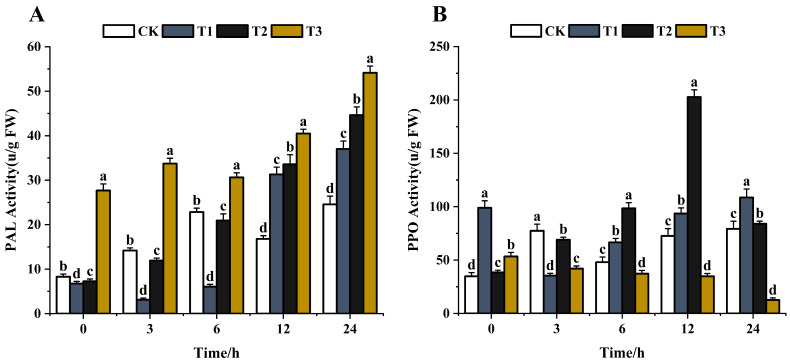
Influence on phenolic metabolism enzyme activity. (**A**) PAL; (**B**) PPO. T1–T3 represent 200 μg/mL of chitosan, difenoconazole, and CS-DIF microcapsules, respectively. Significant differences were determined using one-way ANOVA and Duncan’s multiple range test, as indicated with different letters in the same column of different groups at the *p* < 0.05 significance level.

PAL is the first enzyme in the phenylpropanoid biosynthesis pathway that plays a crucial role in the production of phenolics and phytoalexins [62,63]. The phenolic substances are involved in disease resistance via the phenylpropanoid pathway after various elicitor treatments [64,65]. The effects of the same concentration (200 μg/mL) of chitosan, difenoconazole, and CS-DIF microcapsules on the metabolic enzyme activities of phenolic substances in *C. lunata* (0–24 h) are shown in Figure 10. With the extension of treatment time, the PAL activity showed an overall increasing trend after treatment with difenoconazole and CS-DIF microcapsules compared with the control strain. The enzyme activities increased by 81.7% and 120.4%, respectively. The PAL activity initially decreased following chitosan treatment and then increased, showing a rise of 50.7% compared with the control in 24 h. The results showed that chitosan, difenoconazole, and CS-DIF microcapsules could not enhance the antifungal activity of strains by decreasing the PAL activity. The PPO activity in the control strain generally increased with time, rising by 127.6% in 24 h (Figure 10A). After chitosan treatment, the PPO activity decreased first and then increased, rising by 9.7% in 24 h compared with the control. The PPO activity was defined as the amount of enzyme that caused an increase in absorbance of 0.001/min [66]. The activity increased first and then decreased after difenoconazole treatment, rising by 118.8% in 24 h compared with the control. After treatment with CS-DIF microcapsules, the PPO activity decreased gradually, decreasing by 323.8% in 24 h compared with the control. The results showed that the antimicrobial activity of PPO could be enhanced by continuously reducing the PPO activity of the strain. However, the inhibitory effect of chitosan and difenoconazole on the PPO activity of the strain mainly appeared within 3 h. The subsequent inhibitory activity had little effect on the PPO activity of the strain (Figure 10B). Therefore, the effect of 200 μg/mL) CS-DIF microcapsules focused mainly on the activity of phenol oxidase on the PPO enzyme. The same concentration of chitosan and difenoconazole had little effect on the two phenol metabolism enzymes.

#### 3.4.3. Reactive Oxygen

**Figure 11:**
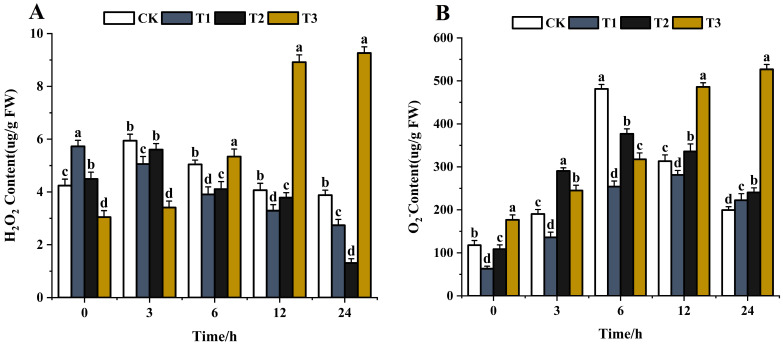
Influence on reactive oxygen species. (**A**) H_2_O_2_; (**B**) O_2_^−^. T1–T3 represent 200 μg/mL of chitosan, difenoconazole, and CS-DIF microcapsules, respectively. Significant differences were determined using one-way ANOVA and Duncan’s multiple range test, as indicated with different letters in the same column of different groups at the *p* < 0.05 significance level.

The reactive oxygen species (ROS) H_2_O_2_ has received much attention in the last decades [67,68]. Sufficient evidence has proven the vital role of H_2_O_2_ in plants under severe environmental conditions, including various biotic and abiotic stresses [69,70]. The ROS content, such as H_2_O_2_ and O_2_^−^, increased when pathogens were treated with exogenous substances [71]. The higher the content of ROS, the greater the impact. With the extension of time (0–24 h), the H_2_O_2_ content gradually decreased after chitosan treatment. The H_2_O_2_ content decreased by 52.2% in 24 h compared with the control (Figure 11A). After difenoconazole treatment, the H_2_O_2_ content first increased and then decreased and was lower by 70.8% compared with the control in 24 h. The H_2_O_2_ content increased gradually after treatment with CS-DIF microcapsules and increased by 204.6% compared with the control in 24 h (Figure 11A). O_2_^−^ is the primary ROS produced by plants during metabolism [72]. The O_2_^−^ content showed an overall increasing trend after treatment with chitosan, difenoconazole, and CS-DIF microcapsules. The contents increased by 11.4%, 20.5%, and 164%, respectively, compared with the control in 24 h (Figure 11B). The ROS results showed that 200 μg/mL chitosan and difenoconazole could enhance the antibacterial activity of strains by increasing O_2_**^−^** content. The antibacterial activity of CS-DIF microcapsules at the same concentration was associated with an increase in H_2_O_2_ and O_2_**^−^** contents. Significant differences were determined using one-way ANOVA and Duncan’s multiple range test, as indicated with different letters in the same column of different groups at the *p* < 0.05 significance level.

## 4. Conclusions

The CS-DIF microcapsules were synthesized by loading difenoconazole into chitosan. The characteristics of the synthesized microcapsules, such as morphology, size, crystal structure, functional groups on the surface, thermal stability, and surface charge, were analyzed using SEM, TEM, XRD, FTIR, TG, and zeta potential. The EE and chemical loading capacity showed that the CS-DIF microcapsules could encapsulate difenoconazole into their unique structure. Compared with difenoconazole, the CS-DIF microcapsules showed prominent controlled-release performance and wettability. Chitosan, difenoconazole, and CS-DIF microcapsules exhibited certain antifungal activity against *C. lunata*, including colony growth inhibition. The influence of microcapsules on mycelium morphology, in vitro inoculation, and several physiological and biochemical parameters were observed. After the CS-DIF microcapsules treatment, SOD, POD, PPO, and CAT were seriously affected in vivo, which then destroyed the integrity of the body cells. In addition, the CS-DIF microcapsules showed much higher antifungal activity compared with an equal concentration (200 μg/mL) of difenoconazole and chitosan. Their synergistic antifungal effect was also proven. The findings not only provided a novel approach for the comprehensive management of plant diseases but laid a foundation for the development of nano pesticides. The transcriptome analysis and in vivo inoculation will be performed to discover the molecular antifungal mechanisms and field application of nano pesticides. Further studies are needed to enhance crop productivity, particularly in maize cultivation.

## Data Availability

The original contributions presented in the study are included in the article, further inquiries can be directed to the corresponding authors.
